# Supplementary material for: Long-term outcome of tibial plateau leveling osteotomy using an antimicrobial silver-based coated plate in dogs
Source: PLoS One. 2022 Aug 12;17(8):e0272555. doi: 10.1371/journal.pone.0272555 (PMC9374240; doi:10.1371/journal.pone.0272555)
Supplement: Attachment — Submitted filename: Response to Reviewers.pdf [file pone.0272555.s004.pdf]

Geoffrey Pagès, DVM
Centre Hospitalier Vétérinaire Aquivet
small animal private practice
19, avenue de la Forêt
33320 Eysines
France

Ashraf M. Abu-Seida
PLOS ONE Academic Editor
PLOS
Carlyle House
Carlyle Road
Cambridge, CB4 3DN
United Kingdom

Eysines, 03.07.2022

Dear Academic Editor,

Please find enclosed our revised manuscript, « Long-term outcome of tibial plateau leveling osteotomy using an antimicrobial silver-based coated plate in dogs ».

Below, you may find the reviewers comments, associated with our answers.
All authors have approved the revised manuscript.

We hope our updates will meet your expectations.

Sincerely,

Dr. Geoffrey Pagès

**Journal Requirements:**
Please review your reference list to ensure that it is complete and correct. If you have cited papers that have been retracted, please include the rationale for doing so in the manuscript text, or remove these references and replace them with relevant current references. Any changes to the reference list should be mentioned in the rebuttal letter that accompanies your revised manuscript. If you need to cite a retracted article, indicate the article's retracted status in the References list and also include a citation and full reference for the retraction notice. : Thank you for the comment. One reference (n°16: Radasch RM: Cruciate surgery options: the good, the bad and the ugly. In: The North American Veterinary Conference; January 20, 2015; Marriott Hotel, Orlando, FL) has been removed previously (as requested by Reviewer #2), and has been replaced with: Rogatko CP, Warnock JJ, Bobe G et al: Comparison of iatrogenic articular cartilage injury in canine stifle arthroscopy versus medial parapatellar mini-arthrotomy in a cadaveric model. Vet Surg 2018;47:6-14. Also, a new reference has been added as N°20: Mangram AJ, Horan TC, Pearson ML et al: Guideline for Prevention of Surgical Site Infection, 1999. Am J Infect Control 1999;27:97-132. The following references has been shifted.

**Additional Editor Comments (if provided):**
Please follow up the format of the journal : To the best of the authors' knowledge, the submitted manuscript follows the format of the journal.
Please respond to all reviewers comments : Corrections have been performed, you may find them below.

**Reviewer #1:**
The authors have addressed my concerns. I maintain (i assume they agree) that a better control population would be far superior, but i accept that is not going to happen. In recognition of the time concern, i recommend publication : Thank you. Indeed, we are sadly unable to provide comparable data concerning complications in the control group, and include additional cases, due to protocol inconstancies previous to the inclusion starting date (January 2017).

**Reviewer #2:**
This manuscript has been appropriately revised. Before completing the manuscript, please recheck the following minor points.

1. You should include the numbers in the BioMedtrix TPLO Curved plate and Synthes TPLO plate groups in the Abstract. : This has been corrected
2. Abstract (Line 39): 7→6?, 31→32? Please final check them (and also others)! : This has been corrected. Others have been checked as well and some mistakes have been corrected in the beginning of the discussion in particular
3. Abstract (Results): Please describe which group results. : This has been done
4. Line 159: "ModTPA was calculated (ReTPA minus PostTPA)"→"ReTPA minus PostTPA (ModTPA) was then calculated" : This has been corrected
5. Results (Line: 210-218): Each data such as age, breed, body weight, affected stifle joint, type of rupture etc. should be listed separately for each group. : This has been added.
6. I found that the cases with combined caudal cruciate ligament rupture was included in this revised manuscript. Did you perform only TPLO surgery in these cases? Or did you perform a combination of extracapsular stabilization technique, etc.? : Indeed, these cases all presented partial tears (the term « partial » has been added in this

revised manuscript), and were treated with only TPLO.

7. Line 261-262: Names of bacteria should be in italics. : This has been corrected, and names of bacterias have been added for the control group

8. Line 282: Another title is preferable (from "Statistical analysis" to another title). You did not change it, although you described "These have been changed" in revision letter. : Sorry, we may have misunderstood your request. We thought the issue was the title of the figures. The paragraph has been deleted, and sentences have been included in the corresponding paragraphs above for better comprehension of the analysis.

9. Line 392: Others limitations→Other limitations? : This has been corrected

10. Figures: "0°" on the vertical axis has not been improved… : This has been corrected converting the files into .tiff images (uploaded separately). We hope the visualization will be correct this time.

**Reviewer #3:**

The authors have revised this manuscript and while improved still needs to better incorporate the control group with thorough analysis and better description of analysis in the materials in methods.

Line 96-100: add the information from lines 109-113 to this region of the paragraph : This has been done

Data analysis: need to add statistical methods for comparison of the population statistics between the biomedtrix and synthes groups for continuous and categorical data, also need to add post hoc power analysis performed : These have been added. Also, this paragraph has been modified to simplify explanations for better comprehension

Line 218-219 : need to expand information of the synthes group similar to the biomedtrix group, add median values and ranges for data of the synthes group and statistically compare between groups using the methodology in the data analysis section. must ensure there is not a bias in one group that would predispose to SSI : This has been done. Also, averages values were modified to median values in the BioMedtrix group in this paragraph and throughout the manuscript. Bias evaluation was commented in the discussion : This was mitigated with the inclusion of the control group, formed by a comparable population. The only statistically significant difference between groups was CrCL lesion type (partial or complete rupture), which is not reported to be a risk factor in SSI development [32].

Line 288-290: add in the post hoc power analysis information contained in the discussion at lines 385-388. this is data : This has been done
