# Supplementary material for: Long-term outcome of tibial plateau leveling osteotomy using an antimicrobial silver-based coated plate in dogs
Source: PLoS One. 2022 Aug 12;17(8):e0272555. doi: 10.1371/journal.pone.0272555 (PMC9374240; doi:10.1371/journal.pone.0272555)
Supplement: Attachment — Submitted filename: Response to Reviewers.pdf [file pone.0272555.s005.pdf]

Geoffrey Pagès, DVM
Centre Hospitalier Vétérinaire Aquivet
small animal private practice
19, avenue de la Forêt
33320 Eysines
France

Ashraf M. Abu-Seida
PLOS ONE Academic Editor
PLOS
Carlyle House
Carlyle Road
Cambridge, CB4 3DN
United Kingdom

Eysines, 19.07.2022

Dear Academic Editor,

Please find enclosed our revised manuscript, « Long-term outcome of tibial plateau leveling osteotomy using an antimicrobial silver-based coated plate in dogs ».

Below, you may find the reviewers comments, associated with our answers.
All authors have approved the revised manuscript.

We hope our updates will meet your expectations, and look forward to your answer as the main author is currently very close to the deadline for credentials submission of his ECVS resident formation (31st July 2022).

Sincerely,

Dr. Geoffrey Pagès

**Review comments to the author:**

Reviewer #2:

This submitted paper (PONE-D-22-10523R2) are appropriately revised in response to the reviewer's remarks. I think that this paper is worthy of acceptance in the PLOS ONE. When you send final manuscript, please recheck the following points.

Line 234-235: caudal cruciate ligament tear→caudal cruciate ligament partial? tear: This has been corrected

Line 260-267: The number of major intraoperative complications is 11 (4.4%) or 9 (3.6%)? Which is correct?: This has been corrected to 9 (3.6%). Also overall intraoperative complications number has been corrected from 17 (6.9%) to 15 (6.0%). This mistake was due to the complications initially considered as major by the authors (osteotomy stabilizing Kirschner wire left in place to maintain adequate stabilization (n=2)), and removed during the revision process. Thank you for highlighting this mistake.

Table 2: Sort the major complications in order of number of occurrences.: This has been corrected
