# Supplementary material for: Long-term outcome of tibial plateau leveling osteotomy using an antimicrobial silver-based coated plate in dogs
Source: PLoS One. 2022 Aug 12;17(8):e0272555. doi: 10.1371/journal.pone.0272555 (PMC9374240; doi:10.1371/journal.pone.0272555)
Supplement: Attachment — Submitted filename: Review PONE_D_22_10523.docx [file pone.0272555.s001.docx]

This paper (PONE-D-22-10523) discusses the outcome including surgical site infection (SSI) after tibial plateau levering osteotomy (TPLO) using the BioMedtrix TPLO Curve^®︎^ plate. This paper provides the interesting data. However, it is concerned the evidence level of this paper is weak to conclude that the antimicrobial silver-based (HyProtect^®︎^) coating plate does not reduce SSI, because of no control group, retrospective style, and a single surgeon study at a single institution. In addition, some revisions are necessary to improve the quality of your manuscript as follows.

Major points:

1. Study design (L.94-95): You should describe the exclusion criteria, in more detail (ex. other orthopedic diseases of affected limb, dermatitis, or infections of other area...).

2. Surgical procedure (L.126): Add details about the type and trade name of sterile wound dressings used in this study because of their impact on the occurrence of SSI.

3. Surgical procedure (L.130-131): The postoperative cefalexin dosage seems a little low. Please recheck it.

4. Radiographic evaluation (L.140): The relationship between the person who performed TPLO surgery and the person who evaluated the radiographs is unclear. Please clarify it.

5. Results: Add the percentage (or number) of partial and complete tears of the cranial cruciate ligament.

6. Results (L201-202): Are all intraoperative minor complications the fibula fractures?

7. Results (L216-217) and Table 2: Are patellar fracture and tibial crest fracture minor complication? Please recheck them.

8. Results (L246): Other titles are preferable (ex. Differences in radiographic measurements between observers or Intraobserver variability).

9. Are the screws inserted in these cases coated with the same coating as the TPLO Curve^®︎^ plates? If not, you need to discuss the effect of non-coating screws on SSI in the Discussion.

10. Discussion (L.305-309): Briefly describe the antimicrobial mechanism of silver-based coated implants.

11. Ref 16 is inappropriate as a reference paper. Please delete it.

Minor points:

1. L.66: 3,6% to 16,5%→3.6% to 16.5%
2. L.166: observers’ mean→observer’s mean?
3. L.333: authors ’opinion→author’s opinion?
4. Figure 1: ”0°” added to vertical axis.
5. Figure 2: ”0°” added to vertical axis.
