# Supplementary material for: Long-term outcome of tibial plateau leveling osteotomy using an antimicrobial silver-based coated plate in dogs
Source: PLoS One. 2022 Aug 12;17(8):e0272555. doi: 10.1371/journal.pone.0272555 (PMC9374240; doi:10.1371/journal.pone.0272555)
Supplement: Attachment — Submitted filename: Response to Reviewers.docx [file pone.0272555.s002.docx]

Geoffrey Pagès, DVM

Centre Hospitalier Vétérinaire Aquivet

small animal private practice

19, avenue de la Forêt

33320 Eysines
France

Ashraf M. Abu-Seida

PLOS ONE Academic Editor

PLOS

Carlyle House

Carlyle Road

Cambridge, CB4 3DN

United Kingdom

Eysines, 10.06.2022

Dear Academic Editor,

Please find enclosed our revised manuscript, « Long-term outcome of tibial plateau leveling osteotomy using an antimicrobial silver-based coated plate in dogs ».

Below, you may find the academic editor and reviewers comments, associated with our answers.

A particular comment, shared by all reviewers, was the absence of a control group in this study. While this was corrected in the revised manuscript, the authors were unable to include such a group with all parameters evaluated in the BioMedtrix™ group, such as TPA, bone healing and intraoperative complications, since medical records of those procedures were inconsistently available, particularly for radiographs.

All authors have approved the revised manuscript.

We hope our updates will meet your expectations.

Sincerely,

Dr. Geoffrey Pagès

Academic Editor:

If applicable, we recommend that you deposit your laboratory protocols in [protocols.io](http://protocols.io/) to enhance the reproducibility of your results. Protocols.io assigns your protocol its own identifier (DOI) so that it can be cited independently in the future. For instructions see: <https://journals.plos.org/plosone/s/submission-guidelines#loc-laboratory-protocols>. Additionally, PLOS ONE offers an option for publishing peer-reviewed Lab Protocol articles, which describe protocols hosted on [protocols.io](http://protocols.io/). Read more information on sharing protocols at <https://plos.org/protocols?utm_medium=editorial-email&utm_source=authorletters&utm_campaign=protocols>.

We deposited the protocols on [protocols.io](http://protocols.io) as requested. You may find them using the following links:

- Anesthetic, analgesic, and antibiotic protocol: [dx.doi.org/10.17504/protocols.io.e6nvwkqm2vmk/v1](https://dx.doi.org/10.17504/protocols.io.e6nvwkqm2vmk/v1)
- Radiographic evaluation: [dx.doi.org/10.17504/protocols.io.36wgq7qekvk5/v1](https://dx.doi.org/10.17504/protocols.io.36wgq7qekvk5/v1)
- Data analysis: [dx.doi.org/10.17504/protocols.io.x54v9yowqg3e/v1](https://dx.doi.org/10.17504/protocols.io.x54v9yowqg3e/v1)

However, we are concerned about the peer-reviewed publication of these protocols, if this may extend the duration of the publication process of this article.

The main author is currently very close to the deadline for credentials submission of his ECVS resident formation (31st July 2022).

Additional Editor Comments:

There are few grammatical issues throughout the manuscript, please correct them and follow up the format of the journal.

Corrections have been made throughout the manuscript for identified grammatical issues, thanks to the help of a native English colleague.

Reviewer #1:

The authors have submitted a simple and clean evaluation and are to be commended. I relatively few comments:

line 53 - change preferred to recommended: This has been changed
line 62 - complications should be complication: This has been changed
line 63 - delete "the most": This has been deleted
line 69 - change claiming to designed for: This has been changed
line 72 - delete designed: This has been deleted
line 74 - change "to protect" into "as providing protection": This has been changed
Line 75 - add "of this study" after objective: This has been added
line 95 - clarify that additional surgery due to complication (explant) was not cause for exclusion. At this early point in the manuscript the point isn't clear.: We added to the previous sentence: Additional procedures due to complications (septic arthritis, implant explantation, late meniscal tear…) were not cause for exclusion.
line 98 (or somewhere before discussion) - the authors need to describe how complications were classified and what exactly constituted a complication. They cover some of this in the discussion but it should be here also. Why is patellar tendinitis listed as both a minor and a major complication?: This has been added in « Complications evaluation ». As a result, patellar tendinitis was classified as a minor complication if no additional treatment was performed and the condition resolved spontaneously, and as a major complication when extended rest and NSAIDs were required to resolve the condition.
line 114 - of should be to: This has been changed
line 144 - ModTPA should be explained as ReTPA minus PostTPA (thus the positive values): This has been added
line 155 - this might be the place to further describe the application of Cook's system: Indeed, comment for line 98 has been added here
line 184 - previous values for ModTPA should be reported (probably in the discussion): This has been added in the discussion
Figures 1, 2, 3 - perhaps better positioned after the section discussing the statistical analysis: Indeed, this was a mistake
line 255 change demonstrates t supports: This has been changed
line 308 - efficiency should be evidence of efficacy: This has been changed

OVERALL - despite the brief discussion in lines 315-324, the major deficiency of this study is the lack of a control group relative to this hospital. The data alluded to should be incorporated into this study, or, preferably, a similar cohort population from previous years using a different plate should be evaluated identically. Obviously there is a variable introduced about time, but this data would still be preferable to the historical/literature comparisons currently presented. This will be annoying, but will dramatically improve the manuscript.: The data have been included in the study. However, a similar cohort population was not achievable, as surgical management and available clinical records were markedly modified before this date.

Reviewer #2:

This paper (PONE-D-22-10523) discusses the outcome including surgical site infection (SSI) after tibial plateau levering osteotomy (TPLO) using the BioMedtrix TPLO Curve®︎ plate. This paper provides the interesting data. However, it is concerned the evidence level of this paper is weak to conclude that the antimicrobial silver-based (HyProtect®︎) coating plate does not reduce SSI, because of no control group, retrospective style, and a single surgeon study at a single institution. In addition, some revisions are necessary to improve the quality of your manuscript as follows.: In this study, two dipl. ECVS surgeons performed the procedures. This was not accurately explained in the manuscript and has been corrected (please see line 91). Also, a control group was formed for SSI analysis.

Major points:
1. Study design (L.94-95): You should describe the exclusion criteria, in more detail (ex. other orthopedic diseases of affected limb, dermatitis, or infections of other area...).: This has been changed to: Dogs with a history of stifle surgery on the affected limb, presented with potential infectious condition (local or generalized dermatitis, prostatitis…), or requiring an additional stifle joint procedure (patellar stabilization…) were excluded. Additional procedures due to complications (septic arthritis, implant explantation, late meniscal tear…) were not cause for exclusion.
2. Surgical procedure (L.126): Add details about the type and trade name of sterile wound dressings used in this study because of their impact on the occurrence of SSI.: This has been added.
3. Surgical procedure (L.130-131): The postoperative cefalexin dosage seems a little low. Please recheck it.: Thank you, this was a mistake. The correct used dosage was 20 mg/kg twice a day.
4. Radiographic evaluation (L.140): The relationship between the person who performed TPLO surgery and the person who evaluated the radiographs is unclear. Please clarify it.: This has been changed to: Two observers (1 Dipl. ECVS and 1 ECVS resident) assessed the anonymized radiographs on a DICOM viewer (Horos™ v3.3.6, horosproject.org). : The Dipl. ECVS observer performed 44% of the procedures.
5. Results: Add the percentage (or number) of partial and complete tears of the cranial cruciate ligament.: This has been added, along with number of caudal cruciate ligament tears: One hundred and sixty-three (66.0%) dogs presented a complete CrCL rupture, and 84 (34.0%) dogs presented a partial CrCL tear. Fifteen (6.1%) dogs presented a caudal cranial cruciate ligament tear.
6. Results (L201-202): Are all intraoperative minor complications the fibula fractures?: Yes, we modified the sentence to: All minor intraoperative complications were fibular fractures, which developed during tibial plateau rotation.
7. Results (L216-217) and Table 2: Are patellar fracture and tibial crest fracture minor complication? Please recheck them.: Yes, as described by Cook and al. (2010), these complications did not require further medical or surgical management, and thus were classified as minor complications. This classification has been detailed in « Complications evaluation ».
8. Results (L246): Other titles are preferable (ex. Differences in radiographic measurements between observers or Intraobserver variability).: These have been changed
9. Are the screws inserted in these cases coated with the same coating as the TPLO Curve®︎ plates? If not, you need to discuss the effect of non-coating screws on SSI in the Discussion.: Thanks for this relevant comment. Indeed, coated screws are not provided in this system. The discussion has been modified.
10. Discussion (L.305-309): Briefly describe the antimicrobial mechanism of silver-based coated implants.): This has been added
11. Ref 16 is inappropriate as a reference paper. Please delete it.: This reference has been replaced by: Rogatko CP, Warnock JJ, Bobe G et al: Comparison of iatrogenic articular cartilage injury in canine stifle arthroscopy versus medial parapatellar mini-arthrotomy in a cadaveric model. Vet Surg 2018;47:6-14

Minor points:
1. L.66: 3,6% to 16,5%→3.6% to 16.5%: This has been corrected
2. L.166: observers’ mean→observer’s mean?: This has been corrected
3. L.333: authors ’opinion→author’s opinion?: This has been removed
4. Figure 1: ”0°” added to vertical axis.: We hope there is no format compatibility issue with the figures. In our version the « 0° » is present for vertical axis.
5. Figure 2: ”0°” added to vertical axis.: This is the same for Figure 2, we also have « 0° » in our version.

Reviewer #3:

This is a manuscript that investigates a newer implant that offers a special coating which in theory should reduce the incidence of surgical site infection. Such plate coatings are being advertised more aggressively, and their effectiveness should be studied. The results of this smaller scale report indicate a similar infection rate to previous studies and thuse the coating does not appear to offer a clinical benefit.
Overall this manuscript could use some editorial assistance as there are some minor grammatical issues interspersed throughout the manuscript.

Line 53- tibial plateau leveling osteotomy.... not plate: We apologize for such a mistake in the first lines of the article. This has been corrected.
Need to define the minor, major, and catastrophic complications. Referencing the cook paper is appropriate, but those definitions still need to be present within the manuscript: This has been added in « Complications evaluation ».
Line 204- cranial tibial artery?: Indeed. This has been corrected.
Line 204- the retention of a K-wire is not considered a complication to this reviewer as its presence is not in a direct response to complication such as tibial tuberosity fracture, it was just left in place for perceived increase in construct stability. This is also questionable given reports exist which indicate a retained K wire does not provide significant increase in stability: This has been modified, together with complication rates in the discussion.
Line 215-222: Need to better define what is major vs. minor complication. For example, a patella fracture is defined as minor which was likely treated conservatively with rest and NSAIDs. Why is patellar tendinitis classified as a major complication when it would have likely also been treated with rest and NSAIDS. The consistency between these issues is not apparent to this reviewer, again clearly defining the criteria used in this report and reason conditions were categorized as major or minor needs to be clarified: Precisions have been added to the description of the complications, both for materials and methods in « Complications evaluation », and in « Postoperative complications » to better explain reasons why specific complications were classified as minor or major.
Line 240- where all SSI confirmed by positive culture? If not then what definition and criteria were used. If culture was used for all then consider summary of the bacterial culture results as it would be interesting to see if there is any difference in bacteria isolates with regard to plate coating: SSI were diagnosed using the definition proposed by Mangram in 1999 (the reference has been added in the manuscript). As a result, bacterial cultures were infrequently performed for superficial and deep SSI resolved with an empirical antibiotic treatment. Culture was positive in only 8 cases (for a total of 12 implant explantations), sadly preventing analysis, particularly to evaluate a difference related to coating.
Line 315-325- this reviewer is uncertain how to feel about this data being contained in the discussion, if it is from the authors' centers then it could be included in the study design and should be added to the material methods and statistical analysis, essentially changing the study design to a retrospective cohort comparison and potentially strengthening the article. This reviewer would recommend major revision to include this information. If not desired, then it would be better to compare study data results to previous reports (i.e historical data) as previous TPLO reports using non-coated implants are abundant and the study results are comparable to reach the same conclusion. As written, the authors attempt to 'backdoor' data that is not fully explained to strengthen their conclusion. A retrospective cohort study design with direct comparison would be best as it improves the strength of study design.: A control group has been formed as suggested.
