# Supplementary material for: Long-term outcome of tibial plateau leveling osteotomy using an antimicrobial silver-based coated plate in dogs
Source: PLoS One. 2022 Aug 12;17(8):e0272555. doi: 10.1371/journal.pone.0272555 (PMC9374240; doi:10.1371/journal.pone.0272555)
Supplement: Attachment — Submitted filename: Review PONE-D-22-10523R1.docx [file pone.0272555.s003.docx]

This manuscript has been appropriately revised. Before completing the manuscript, please recheck the following minor points.

1. You should include the numbers in the BioMedtrix TPLO Curved plate and Synthes TPLO plate groups in the Abstract.
2. Abstract (Line 39): 7→6?, 31→32? Please final check them (and also others)!
3. Abstract (Results): Please describe which group results.
4. Line 159: “ModTPA was calculated (ReTPA minus PostTPA)”→”ReTPA minus PostTPA (ModTPA) was then calculated”
5. Results (Line: 210-218): Each data such as age, breed, body weight, affected stifle joint, type of rupture etc. should be listed separately for each group.
6. I found that the cases with combined caudal cruciate ligament rupture was included in this revised manuscript. Did you perform only TPLO surgery in these cases? Or did you perform a combination of extracapsular stabilization technique, etc.?
7. Line 261-262: Names of bacteria should be in italics.
8. Line 282: Another title is preferable (from “Statistical analysis” to another title). You did not change it, although you described “These have been changed” in revision letter.
9. Line 392: Others limitations→Other limitations?
10. Figures: “0°” on the vertical axis has not been improved...
